# Ultrasonographical Evaluation of the Median Nerve Mobility in Carpal Tunnel Syndrome: A Systematic Review and Meta-Analysis

**DOI:** 10.3390/diagnostics12102349

**Published:** 2022-09-28

**Authors:** Yu-Ting Huang, Chii-Jen Chen, You-Wei Wang, Po-Lin Peng, Yan-Ting Luo, Yi-Shiung Horng

**Affiliations:** 1 Department of Medical Education, Taipei Tzuchi Hospital, Buddhist Tzuchi Medical Foundation, New Taipei City 23142, Taiwan; 2 Department of Computer Science and Information Engineering, Tamkang University, New Taipei City 25137, Taiwan; 3 Department of Computer Science and Information Engineering, National Taiwan University, Taipei 10617, Taiwan; 4 Department of Physical Medicine and Rehabilitation, Taipei Tzuchi Hospital, Buddhist Tzuchi Medical Foundation, No. 289, Jianguo Rd., Xindian Dist., New Taipei City 23142, Taiwan; 5 Department of Medicine, Tzu Chi University, Hualien 97004, Taiwan

**Keywords:** carpal tunnel syndrome, median nerve, nerve displacement, ultrasound images, systematic review, meta-analysis

## Abstract

Diagnostic ultrasound is widely used for evaluating carpal tunnel syndrome (CTS), an entrapment neuropathy of the median nerve (MN). Decreased mobility of the MN inside the carpal tunnel has been reported in CTS, and various methods have been used to evaluate MN mobility; however, there is still no conclusive understanding of its connection with CTS. The purpose of this study is to conduct a systematic review and meta-analysis of the current published literature on ultrasonographic evaluations of transverse and longitudinal MN displacement and to identify the relationship between MN mobility and CTS. This study was conducted in accordance with the 2020 PRISMA statement and the Cochrane Collaboration Handbook. Comparative studies that investigated differences in MN displacement between CTS patients and healthy controls were retrieved by searching the Cochrane Library, Embase and PubMed. A total of 15 case–control studies were included. Nine of 12 studies evaluating transverse MN displacement and 4 of 5 studies evaluating longitudinal MN gliding showed that the MN was less mobile in CTS patients than in healthy subjects. Despite the large heterogeneity among the 15 included studies, this systematic review and meta-analysis provide evidence that the mobility of the MN is significantly reduced in both transverse and longitudinal planes in CTS patients compared to healthy controls. Five of the 15 included studies reported that a decrease in transverse or longitudinal MN displacement in CTS was correlated with clinical symptoms or with severity as measured by a nerve conduction study (NCS).

## 1. Introduction

**Figure 1:**
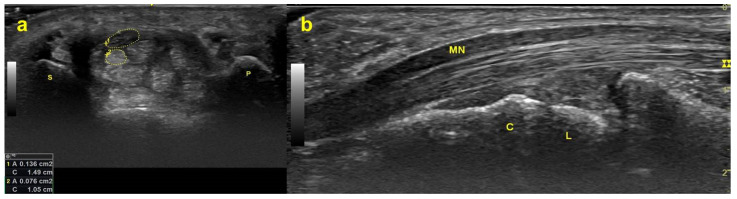
Ultrasonograms captured at the distal wrist crease level. (**a**) MN (structure enclosed by the upper dotted line) and FDS tendon (structure enclosed by the lower dotted line) in the carpal tunnel on the transverse plane. (**b**) MN and FDS tendon on the longitudinal plane. Abbreviations: P = pisiform bone; S = scaphoid bone; MN = median nerve; FDS = flexor digitorum superficialis; L = lunate bone; C = capitate bone.

Carpal tunnel syndrome (CTS) is an entrapment neuropathy of the median nerve (MN) inside the carpal tunnel, which is an osteofibrous canal framed by the transcarpal ligament as the roof and the carpal bones as the floor (Figure 1). The MN and nine flexor tendons (the flexor pollicis longus (FPL), the four flexor digitorum superficialis (FDS), and the four flexor digitorum profundus (FDP)) are surrounded and closely connected by subsynovial connective tissue (SSCT), and they travel through the carpal canal together [1] forming a gliding unit [2].

The diagnosis of CTS is mainly based on clinical manifestations (pain and paresthesia over 1–3 digits and the radial site of the ring finger), physical examinations such as the Tinel and Phalen test, and electrophysiological studies. Although electrophysiological studies, including nerve conduction studies (NCSs) and electromyography (EMG), have been used long-term as gold standards in diagnosing CTS, they are uncomfortable for subjects, and the reported false negative or positive rate is up to 10–15% [3,4,5,6]. Therefore, ultrasonography has been applied to diagnose CTS in recent decades owing to its cost-effectiveness and accessibility [7,8].

For example, Buchberger, et al. [9] first described four features in sonographic images in CTS patients: (1) an increased cross-sectional area (CSA) of the MN at the level of the pisiform and hamate bone, (2) an increased swelling ratio (ratio of the CSA of the MN at the pisiform level to the distal radius level), (3) an increased flattening ratio, defined as the ratio of the length of the long to short axis of the MN, and (4) significant palmar bowing of the flexor retinaculum. Elsaman, et al. [10] introduced a diagnostic criterion for CTS—the depth of the carpal tunnel (DCT), which is defined by the distance from the surface of the flexor retinaculum to the highest point of the capitate bone. To accommodate individual anthropometric variability, the wrist:forearm ratio (WFR), wrist–forearm difference (WFD), median-to-ulnar nerve ratio (MUR) and median-to-ulnar nerve difference (MUD) were also suggested as diagnostic criteria for CTS [11,12,13,14,15]. In addition, increased intraneural vascularity of the MN was also reported in patients with CTS [16,17]. Gonzalez-Suarez et al. [11] revealed that combining the WFD obtained by grayscale sonography and intraneural hypervascularity obtained by color Doppler could provide better sensitivity of up to 98.1%. Some researchers [18,19] further utilized elastography to observe the changes in the nerve environment and tissue elasticity. Kluge et al. [16] also described the changes in echogenicity as another sonographic criterion for CTS. In healthy wrists, the hypoechoic nerve fascicles were enclosed by hyperechoic perineurium, forming a honeycomb-like structure. However, the MN assumes a more hypoechoic pattern in sonographic images as it undergoes edematous changes after mechanical compression in CTS. These edematous changes cause ischemia–reperfusion injury [20]; therefore, the MN becomes swollen and less echogenic in sonograms.

On the other hand, many researchers have tried to use dynamic ultrasonographic studies to evaluate gliding of the MN and flexor tendons [16,21,22]. Since the MN, flexor tendons and SSCT are exposed to repetitive biomechanical compressive and shear stress force during motion of the upper extremities, the gliding of the MN might be reduced when it is sandwiched between tensed tendons and fibrotic SSCT [23,24,25,26,27,28]. Although some researchers have focused on evaluating longitudinal MN gliding [29,30,31,32,33,34,35] and some have focused on transverse MN displacement [1,20,21,29,31,36,37,38,39,40,41,42,43,44,45,46,47,48,49], there are still inconsistent results between studies. Moreover, neither a standard measurement method nor definite criteria for MN displacement have been established for diagnosing CTS. Therefore, the purpose of this systematic review and meta-analysis is to compare the MN displacement between CTS patients and healthy controls and to identify the relationship between MN mobility and CTS. We hypothesize that MN mobility is decreased in CTS patients and is correlated with NCS severity.

## 2. Materials and Methods

### 2.1. Information Sources and Search Strategy

This systematic review was conducted in accordance with the Preferred Reporting Items for Systematic Reviews and Meta-Analyses (PRISMA) 2020 statement [50] and the Cochrane Collaboration Handbook [51]. This study was registered through PROSPERO (registration number: CRD42022310976). The Cochrane Library, Embase and PubMed were chosen as three major electronic databases and were searched from inception to 15 March 2022. The keywords “carpal tunnel syndrome”, “median nerve”, “ultrasound”, and “nerve gliding” (or “nerve sliding”, or “nerve excursion”, or “nerve deformation”, or “nerve displacement”) were searched in these databases with different strategies (Supplementary data, S1). Two authors independently conducted the search process, study selection, and data extraction. Any discordance was resolved by consensus between the two authors and the corresponding author.

### 2.2. Study Selection

This study followed the PRISMA guidelines. The study population consisted of patients with electrodiagnostically confirmed CTS, and the control group consisted of healthy volunteers. The main interventional tool was an ultrasound, and transverse displacement and longitudinal gliding of the MN were the outcomes of interest.

Comparative studies that investigated the differences in MN displacement between CTS patients and healthy subjects with ultrasound were included in this systematic review and meta-analysis. Exclusion criteria included studies lacking electrodiagnostic confirmation in the CTS group or without quantifying MN gliding. Systematic reviews, meta-analyses, narrative reviews, articles that only had an abstract, case reports/series without a control group and studies that only assessed MN gliding in healthy subjects were also excluded.

### 2.3. Data Collection

**Figure 2:**
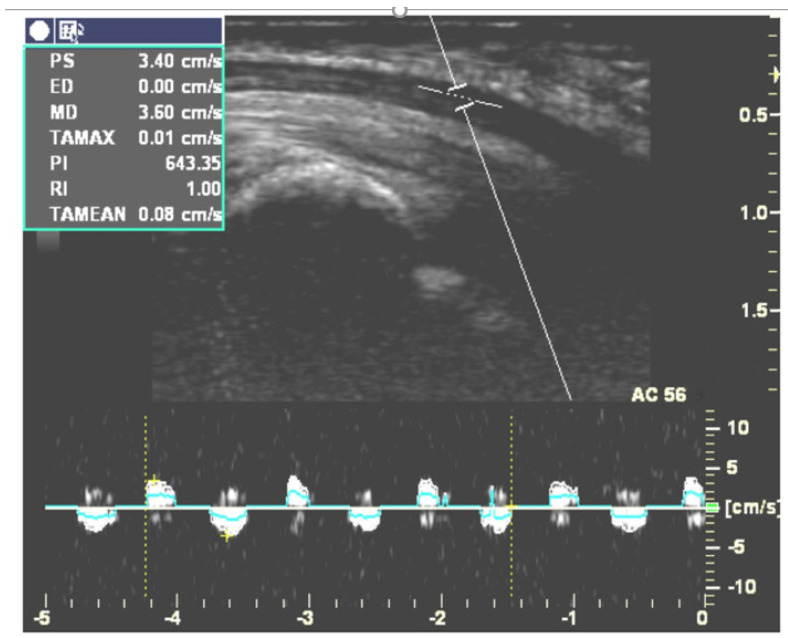
Doppler waveforms for measuring longitudinal MN gliding during active flexion and the extension cycle of the index finger at a speed of one time per second. (This figure was adapted from Liu et al. [33]).

Two independent researchers collected the relevant data from each eligible article, including the authors, published year, average age, sex distribution, number of wrists in each group, diagnostic reference for the CTS group, severity of the CTS group, sites of ultrasonic measurement, and hand or finger postures and movements while performing ultrasonography. Information regarding the ultrasonic analysis methods including speckle tracking algorithm, cross-correlation (CC) algorithm, multilevel block-sum pyramid (BSP) integrate algorithm, duplex Doppler (Figure 2) and parameters (actual MN displacement distance, MN displacement in two directions, normalized displacement, etc.) were also retrieved.

Speckle tracking is a traditional template matching algorithm measuring the similarity between blocks in image processing. It calculates the similarity information as an absolute difference for each block. The CC algorithm calculates the correlation between each pixel to improve tracking accuracy. The multilevel block-sum pyramid integrates algorithm (BSP) is based on CC and the concept of template matching. It uses multiple blocks for each kernel from different scales and calculates the average vector of all correlation-weighted kernels to track the MN movement as a similarity measure [52,53].

### 2.4. Study Risk of Bias Assessment

The Newcastle–Ottawa Quality Assessment Scale (NOS) was used to evaluate the risk of bias for each included study. The NOS is a tool for assessing the quality of nonrandomized studies according to the study design and content. A star system was adopted in this article for judging eight items of three broad perspectives, including (1) the selection of the study groups; (2) the comparability of the groups; and (3) the exposure or outcome of interest. The star system scored each item as 0, 1, or 2 stars and yielded a total of 0 to 9 stars. In the selection section, studies with an adequate definition for CTS, consecutive or obviously representative cases and community controls, or controls without a history of CTS could be awarded a star. In the comparability section, studies could be scored as 1 to 2 stars if confounding factors were well controlled. In the exposure section, a star could be given for studies with secure records, blinded interviews, and the same response rate or enrollment methods for both CTS and control groups. The quality of the included studies was appraised by two independent researchers using the star system of the NOS. Any discrepancy was resolved by discussion and consensus.

### 2.5. Statistical Analysis

The standardized mean difference (SMD) of transverse or longitudinal MN displacement between CTS and control groups comprised the measurement outcome. In the transverse plane, the data were extracted from the actual MN displacement or the MN displacement at the DP and RU axes during the motions of index finger flexion, metacarpophalangeal joints flexion or hand grasp. In the longitudinal plane, the data were extracted from longitudinal MN gliding during the motions. A random effect model was adopted for pooling SMDs of included studies. The statistical analyses were performed by Comprehensive Meta-Analysis (CMA) software, version 3 (Biostat, Englewood, NJ, USA). The heterogeneity between included studies was analyzed by the I-squared test. The I-squared value above 75% indicates moderate to high heterogeneity between included studies. The I-squared value between 50% to 75% indicates mild heterogeneity, while the I-squared value below 50% indicates low or no heterogeneity. The potential publication bias was examined by Funnel plots and Egger’s test. Sensitivity analysis was performed by “leave-one-out” method. It is an iterative procedure in which one trial is left out and a meta-analysis is performed on the remaining subset of the studies at each iteration. This analysis shows how each individual study affects the overall estimate of the rest of the studies. 

## 3. Results

### 3.1. Study Selection

**Figure 3:**
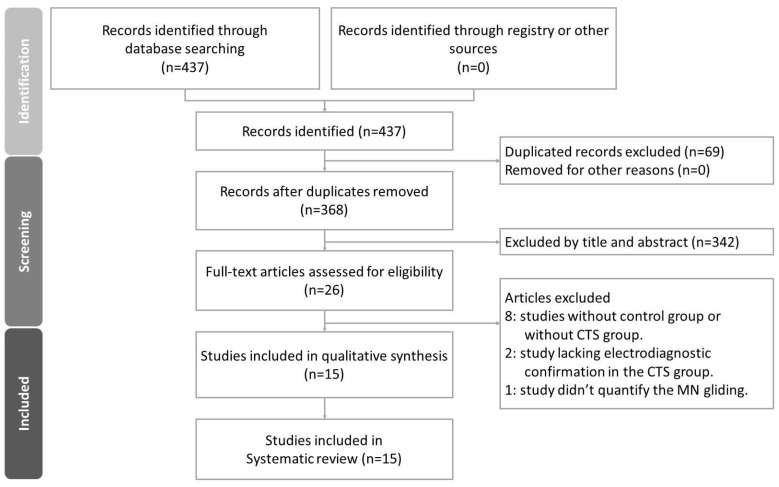
Study selection flow chart.

A detailed selection flow chart is shown in Figure 3. A total of 437 articles were retrieved from the literature search. There were 69 duplicates between the three databases. After removing the duplicates, 368 articles remained and were screened by two researchers independently according to the titles and abstracts. Twenty-six articles then underwent full-text screening. Among them, 8 studies without a control group or without a CTS group were excluded. Four studies lacking electrodiagnostic confirmation in the CTS group and one study that did not quantify MN displacement were further excluded. Finally, 15 studies fulfilling the selection criteria remained in this systematic review. Any discrepancy was resolved by discussion and consensus between authors.

### 3.2. Study Characteristics

A total of 15 case–control studies (513 wrists with CTS and 433 healthy wrists) were enrolled in this systematic review (Table 1). The mean age of the participants was between 41 and 69 years old, and there were more females than males. Some studies [20,31,39,42,43,44] further stratified CTS patients into different severity groups. The methods of ultrasonic image analysis varied between the studies. In most studies, subjects were asked to move their fingers actively, and an ultrasound probe was placed at the proximal carpal tunnel or forearm level [29,44], but in some studies [29,37,54], the subject’s finger or wrist was passively moved by a device. Among the 15 included studies, 2 studies [29,31] measured both transverse and longitudinal gliding of the MN, 10 studies [20,36,37,38,39,41,42,43,44,54] evaluated transverse MN displacement only and the other 3 studies [30,32,33] evaluated longitudinal MN gliding only. Because of heterogeneity, it was difficult to directly compare the data obtained from the different studies or perform a meta-analysis.

### 3.3. Risk of Bias in Studies

The results of the NOS quality assessment are shown in Table 2. Lower scores were mostly awarded due to a lack of controlling for confounding factors or a lack of a blinding procedure.

### 3.4. Results of Syntheses

The baseline characteristics are described in detail in Table 1.

#### 3.4.1. Transverse MN Gliding

The 12 studies targeting transverse MN displacement are shown in Table 3.

There was wide heterogeneity in the protocols and parameters adopted to evaluate transverse MN displacement among the studies. Regarding the methods of measuring MN displacement, some authors [20,31,36,37,43,44] calculated the actual displacement distance of the MN, and some [29,38,40,41,42] measured MN displacement in two directions, including along the dorsopalmar (DP) and radioulnar (RU) axes, while Hara et al. [54] quantified the transverse MN displacement using the summed motion area during a whole motion cycle.

Most studies measured the actual transverse MN displacement distance, which is calculated directly as the distance between the MN on the first and last recorded ultrasonic images. Among the studies that measured the actual transverse MN displacement distance, Nakamichi and Tachibana [37], Kuo et al. [20] and Fan et al. [44] concluded that the CTS group had a smaller actual MN displacement distance at the proximal carpal tunnel during index finger [37] or all fingers [20,44] flexion. In contrast, Wang et al. [36] and Filius et al. [31] found no significant differences between CTS patients and healthy controls. Regarding wrist motions, Wang et al. [36] revealed a smaller actual displacement distance in the CTS group during wrist flexion and ulnar deviation regardless of whether the fingers were flexed or extended.

**Figure 4:**
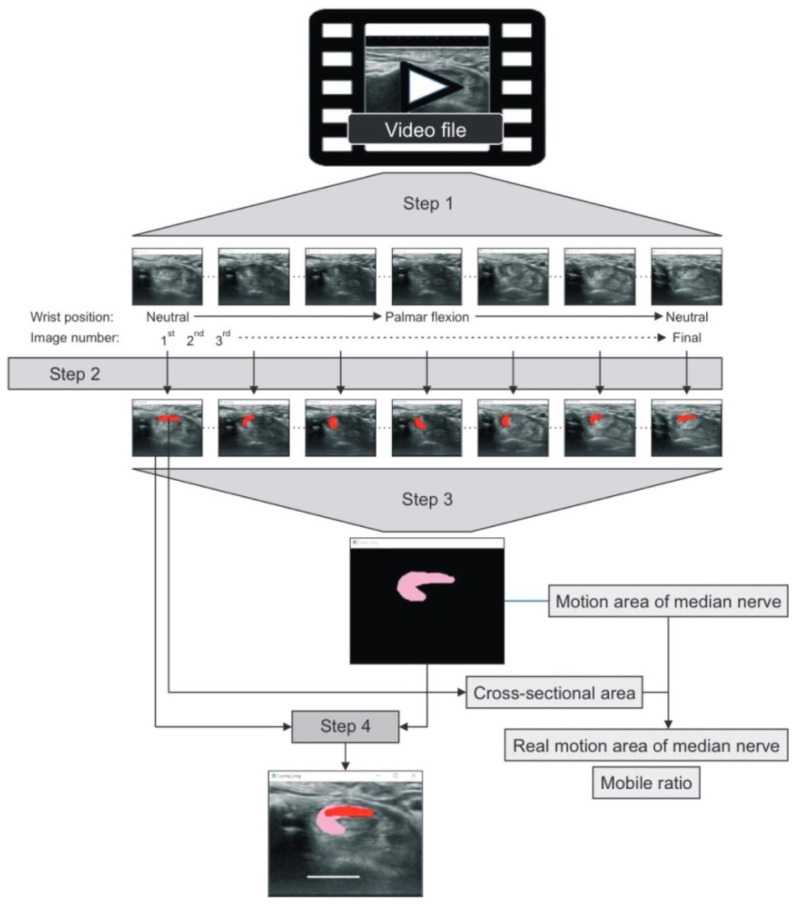
The algorithm for measuring the motion area of the MN was adopted from Hara et al. [54] Images were captured frame by frame from a video file (Step 1), and the cross-sectional area (CSA) of the MN was marked in red (Step 2). The CSAs of the MN from the first to the final images were overlaid to form the motion area of the MN (MAMn) (in pink, Step 3). The real motion area of the MN (RMMn) was defined by subtracting the CSA of the MN from the MAMn, while the mobile ratio (MR) was defined by dividing the MAMn by the CSA of the MN (Step 4).

Moreover, Hara et al. [54] quantified the transverse MN displacement using the summed motion area of the MN (MAMn) during a whole motion cycle. They [54] further subtracted the CSA of the MN from the MAMn (defined as the real motion area, RMMn) and divided the MAMn by the CSA of the MN (defined as the motion ratio, MR) (as shown in Figure 4). In their study, a significantly smaller RMMn and MR were observed in the CTS group than in healthy controls.

The other studies evaluated transverse MN displacement in two directions along the DP and RU axes. Yoshii et al. [39] found that patients with CTS had significantly decreased MN displacement along the DP axis but not along the RU axis during flexion of all five fingers. van Doesburg et al. [38] and Kang and Yoon [42] found that the MN moved significantly less in the dorsal and radial directions during thumb flexion in the CTS group. More interestingly, the MN moved toward the ulnar-palmar side in the control group, while it moved toward the dorsoradial side in the CTS group during thumb flexion. In addition, Kang and Yoon [42] found a significant decrease in MN displacement in the CTS group during index finger flexion along the RU axis and third finger flexion along the DP axis. When subjects gripped with index, middle, ring, and small finger flexion, significantly smaller MN displacement was also noted along both the DP and RU axes. In contrast, van Doesburg et al. [38] revealed no significant difference between the CTS and control groups during these motions. Three studies [20,42,43] investigated the relationship between transverse MN displacement and CTS severity. Kang and Yoon [42] revealed that transverse MN displacement was negatively correlated with the CSA of MN. Kuo et al. [20] and Park et al. [43] also noted a negative association between transverse MN displacement and NCS severity in CTS.

#### 3.4.2. Longitudinal MN Gliding

Five studies targeting longitudinal gliding of the MN are described in Table 4. Two [30,33] of them used duplex Doppler to evaluate longitudinal gliding of the MN by adjusting the Doppler sample volume indicator to lie within the MN (Figure 2). They recorded the Doppler waveform while subjects continuously moved their fingers and calculated the area under the Doppler waveform in the velocity-time integral (VTI) spectrum. This area represented the amount of longitudinal MN gliding during each cycle of finger movement [55]. Hough et al. found that CTS patients had less MN gliding than healthy controls during elbow extension but not during elbow flexion [30]. Liu et al. [33] measured longitudinal MN gliding in both the neutral and 30 degree wrist extension positions. Their results revealed that the ratio of the gliding of the MN to the flexor tendon was significantly smaller in CTS patients than in healthy volunteers. They also found that gliding of the MN was significantly increased while extending the wrist joint to 30 degrees in CTS patients [33]. The other three studies recorded B-mode dynamic ultrasonography while subjects performed the target movements and used a cross-correlation algorithm or speckle-tracking algorithm to evaluate MN gliding frame-by frame [29,31,32]. For example, Erel et al. [29] analyzed longitudinal MN gliding at the forearm level during passive motion of the metacarpophalangeal joint, but their results did not demonstrate a significant difference in MN gliding between the CTS and control groups. In contrast, Filius et al. [31] demonstrated less longitudinal MN gliding in CTS patients. Filius et al. also published another article [32] exploring the relationship between tendon excursion velocity and longitudinal MN gliding. They found that the higher the moving velocity of the fingers, the greater the MN glided; moreover, MN gliding was reduced in the CTS group whenever fingers were moving at high or low speed.

Two studies [31,33] also investigated the relationship between longitudinal MN gliding and CTS severity. Filius et al. [31] revealed a negative correlation between the ratio of median nerve excursion to flexor tendon excursion and CTS severity. Liu et al. [33] further demonstrated that this ratio was weakly to moderately correlated with symptom severity, functional status, mid-palm latency, distal median motor and sensory latency.

### 3.5. Results of Meta-Analysis

#### 3.5.1. Transverse MN Displacement

**Figure 5:**
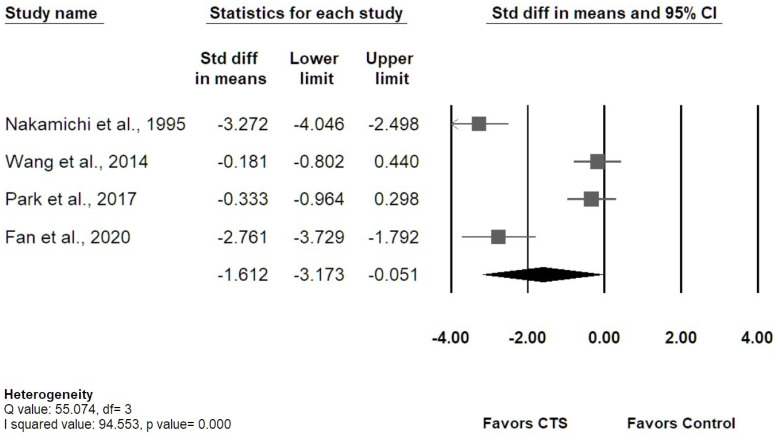
The overall transverse MN displacement during index finger flexion or hand grasping in the CTS group compared with the control group.

**Figure 6:**
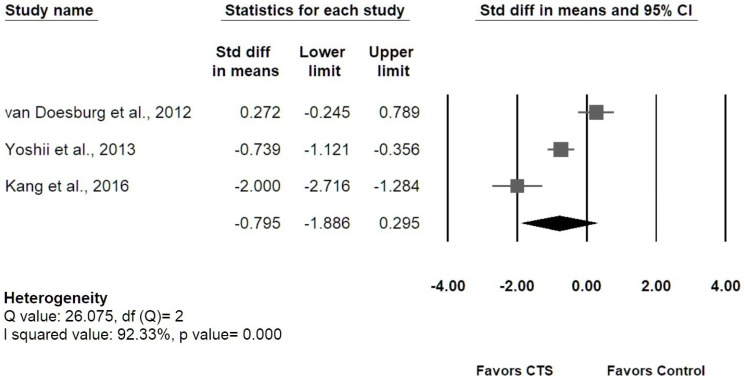

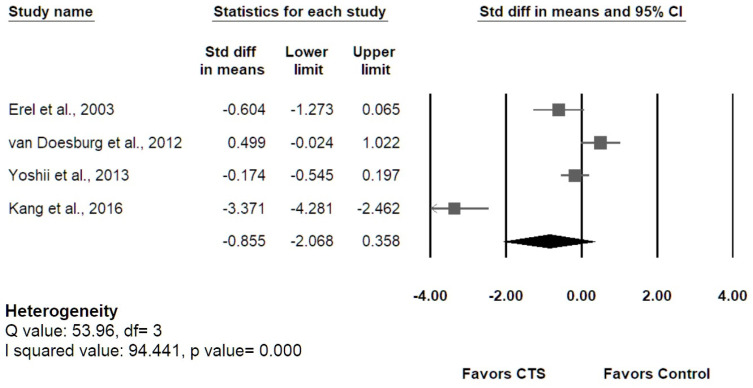
The overall transverse MN displacement at the dorsopalmar axis (graph on the **upper**) and at the radioulnar axis (graph on the **lower**) during the index finger flexion or hand grasping in the CTS group compared with the control group.

Among the 12 studies measuring transverse MN displacement, four [36,37,43,44] studies were enrolled for meta-analysis regarding actual MN displacement, and the results revealed that patients with CTS had smaller MN displacement than healthy controls, with overall SMD of −1.612 (95% confidence interval [CI]: −3.173 to −0.051) (Figure 5). The I-squared value was 94.55% (*p* value = 0.00), which indicated a high heterogeneity. Three studies [38,39,42] investigating MN displacement at DP or RU axis were enrolled for meta-analysis. No significant difference in MN displacement between groups was found in both DP and RU axes (Figure 6). The results of Egger’s tests for the above three meta-analyses revealed no significant publication bias (*p* = 0.17; 0.74; 0.46, respectively), and the Funnel plots were shown in the Supplementary of Figures S1–S3. 

#### 3.5.2. Longitudinal MN Gliding

**Figure 7:**
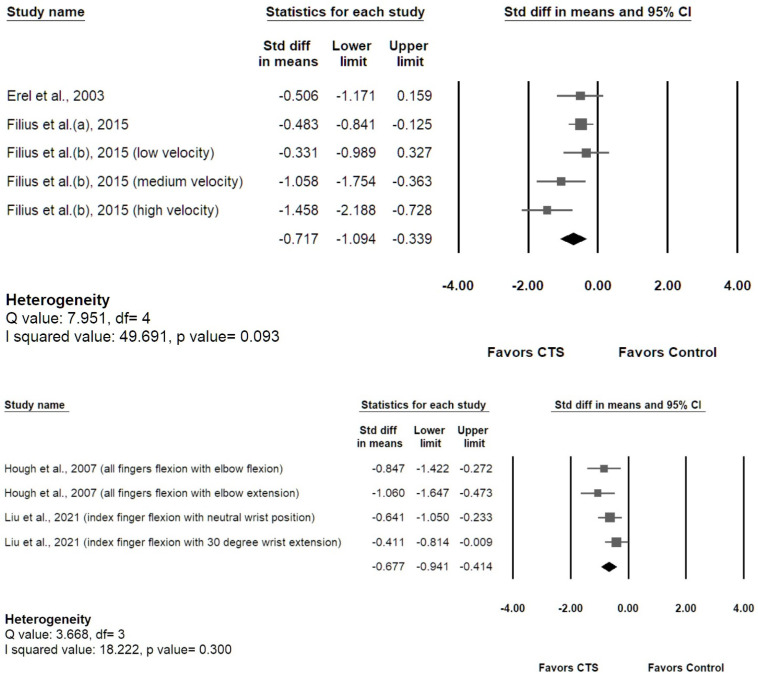
The overall longitudinal gliding of the MN in the CTS group compared with the control group. Pooled result of studies using speckle tracking method (graph on the **upper**). Pooled results of studies using duplex Doppler waveform method (graph on the **lower**).

As shown in Figure 7, the meta-analysis of the five studies [29,30,31,32,33] investigating longitudinal MN gliding revealed that patients with CTS had smaller MN gliding than healthy controls (speckle tracking method: SMD −0.717, 95% [CI] −1.094 to −0.339; duplex Doppler method: SMD −0.677, 95% [CI] −0.941 to −0.414). Low heterogeneity was revealed by I-squared (speckle tracking method: 49.69%, *p* value = 0.093; duplex Doppler method: 18.22%, *p* value = 0.300). The Egger’s tests revealed no significant publication bias (*p* values were 0.33 and 0.10, respectively), and the Funnel plots are provided in Supplementary of Figures S4 and S5. 

#### 3.5.3. Sensitivity Analysis

“Leave-one-out” evaluation was used to assess the stability of the estimated measures. The results showed the pooled point estimates lay within the 95% CI of the overall pooled effect (Supplementary Figures S6–S9). Therefore, the pooled SMDs of actual transverse displacement, displacement in RU and DP axes, and longitudinal gliding of the MN revealed no significant influence on the overall analysis by any individual study, indicating consistency in the pooled results.

## 4. Discussion

A total of 15 case–control studies were included in this systematic review and meta-analysis, with two planes (transverse and longitudinal) and two ultrasound modes (traditional grayscale and duplex Doppler method) used for the assessment of MN mobility. Two outcome indicators (actual transverse displacement and transverse displacement in the DP and RU axes) were used to assess the transverse mobility of the MN, and longitudinal MN gliding was used to assess the longitudinal mobility of the MN. Despite the large heterogeneity among the 15 included studies regarding hand motion and upper limb position during ultrasonography, the results of this systematic review and meta-analysis support that the mobility of the MN is significantly smaller in CTS patients than healthy controls when using the indicators of actual transverse displacement and longitudinal gliding. However, no significant decrease in MN mobility was observed in CTS patients while using the transverse MN displacement at DP and RU axes as indicators. These pooled results revealed that the “actual MN displacement” might better reflect the real MN mobility in the transverse plane rather than measuring at DP or RU axis.

The MN and the surrounding tissue are organized into concentric layers and form a gliding unit [44]. MN mobility is considered a reflection of the degree of fibrosis of the epineurial and perineurial tissue of the MN and SSCT in CTS, and ultrasonographic evaluation enables the quantification of the kinematics of the MN inside or outside the carpal tunnel [43,49]. The pathogenesis of CTS includes tenosynovitis of the flexor tendons, adhesion inside the carpal tunnel and increased carpal tunnel pressure. Repetitive biomechanical compressive and shear stress contributes to noninflammatory fibrosis of the SSCT and even the epineural and perineural tissues of the MN [43]. Moreover, fibrosis of the SSCT further interferes with the smooth gliding of the median nerve and flexor tendons, which induces repetitive trauma even during normal movement of the hand [56].

In healthy subjects, the MN is presumed to glide around the common flexor sheath and sink smoothly into the gap between the FPL and the common flexor tendon sheath. However, Hara et al. [54] observed that the MN in CTS patients failed to move to a deeper layer, possibly because of the closure of the potential space between the flexor tendons resulting from fibrosis of the surrounding tissues. Moreover, the space between the FPL and common flexor tendon sheath may become insufficient as fibrosis progresses. In that event, the MN will be confined just beneath the flexor retinaculum and create a vicious cycle of decreased MN mobility, which can further increase the risk of developing tenosynovitis and/or aggravating a preexisting condition. However, in a study by Filius et al. [31], decreased MN mobility was observed only in the longitudinal direction and not in the transverse plane. During finger motion, the MN passively glided under indirect traction from the flexor tendons via the SSCT. A previous study found that in some CTS patients, the SSCT was not only fibrotic but also ruptured, resulting in dissociation between the SSCT and the tendons [56]. This phenomenon might explain why transverse displacement was not decreased as much as longitudinal gliding: the MN is no longer firmly attached to the flexor tendons and moving together with them.

### 4.1. Summary of Evidence

Among 12 studies measuring transverse MN displacement, three [20,37,48] of six studies [20,31,36,37,43,44] measured the actual displacement distance of the MN and concluded that the transverse MN displacement was significantly smaller in CTS patients during index finger [37] or all finger [20,44] flexion. Four studies [38,39,41,42] focused on transverse MN displacement along the DP axis, and five studies [29,38,39,41,42] examining transverse MN displacement along the RU axis revealed heterogeneous results. For example, along the DP axis, two [41,42] of four studies found a significantly smaller MN displacement in CTS patients, while one study [39] revealed that the difference was not statistically significant, and another study [38] even noted a greater MN displacement in the CTS group, although the difference did not reach statistical significance. The same discrepant results were also noted in the five studies evaluating the RU axis [39,41,42]. Other than the 11 studies mentioned above, one study [54] adopted a new measurement method, the “motion area” of the MN and found that it was smaller in the CTS group. The results of the five studies evaluating longitudinal MN gliding showed less discrepancy. Only one study did not show a statistically significant decrease in MN gliding, while the other four studies all demonstrated decreased MN gliding even at various wrist [33] and elbow [30] positions with different motion velocities [32]. On the other hand, five of the 15 included studies demonstrated that transverse [20,42,43] and longitudinal [31,33] MN displacement in CTS were negatively correlated with clinical symptoms or NCS severity.

### 4.2. Risk Assessment of Evidence

The risk of bias was assessed by the Newcastle–Ottawa Quality Assessment Scale (NOS), which used a star system (0 to 9 stars) to judge eight items covering three areas. As shown in Table 2, all 15 included studies had rigorous definitions of selection criteria and received a full score (4 stars) in the area of selection. However, in the category of comparability, or controlling for important factors, only 4 studies [29,37,42,44] scored 2 stars by effectively controlling for confounding factors including age and gender. Of the three items in the category of exposure or outcome of interest, only 3 studies [30,33,37] scored 3 stars. The other 12 studies failed to gain a star for the item “ascertainment of exposure” due to a lack of independent or blind assessment. Overall, one study [37] scored 9 stars, five studies [29,30,33,42,44] scored 8 stars, and 9 studies [20,31,32,36,38,39,41,43,54] scored 7 stars. The quality of the included studies was ranked between moderate and good according to the NOS.

### 4.3. Heterogeneity between Studies

There was great variability among the 15 included studies regarding the position of the subjects’ shoulder, elbow and wrist joints during ultrasonography. Although most studies [20,29,31,32,37,38,39,42,43,44] measured MN displacement with the wrist joint in a neutral position, two studies [30,33] evaluated MN displacement in the 30-degree wrist extension position. Wang et al. [36] and Nanno et al. [41] obtained measurements not only with the wrist joints in the neutral position but also with them in flexion, extension and deviated radially and ulnarly, while Hara et al. [54] mainly focused on MN displacement during flexion of the wrist joint. Some studies [29,37,42,43,44,54] fixed the elbow in full extension, while other studies [30,31,32,39,41] asked the subjects to flex their elbows at 45 [41], 60 [32], 90 [30] or 120 [31] degrees. A few studies mentioned that they kept the shoulder in a neutral [36,41,43,44] position, slightly abducted or at 45 or 90 degrees of abduction with forearm supination [29,31,36,39,41,42,43,44,54]. Since the MN travels through the shoulder, elbow and wrist joint, the tension and mobility of the MN inside the carpal tunnel will be influenced by the position of the adjacent joints. This might partially explain the heterogeneous results among the 15 included studies.

Moreover, there was variability in the measurement site among the studies. Although most studies measured MN displacement around the proximal carpal tunnel, Erel et al. [29] performed ultrasonography 5 to 15 cm proximal to the distal wrist crease and found no significant difference in longitudinal MN gliding between CTS patients and healthy subjects. Since entrapment of the MN occurs in the carpal tunnel but not at the proximal forearm, this might explain why the results of their study were inconsistent with those of other studies. However, Fan et al. [44] measured transverse MN displacement in both the proximal carpal tunnel and mid-forearm, and they found a significantly smaller actual transverse MN displacement distance at both levels.

### 4.4. Variability of Measuring Methods

Among the articles measuring MN displacement in the transverse plane, some studies used an anatomical structure as a fixed reference point to measure displacement of the MN. For example, Nakamichi and Tachibana [37] monitored the MN displacement distance using the ulnar artery as the point of reference. Nanno et al. [41] represented the MN as a coordinate point using the midpoint between the apex of the ridge of the trapezium and the apex of the hook of the hamate as references. Kang and Yoon [42], Park et al. [43] and Fan et al. [44] used the scaphoid bone and the tubercles of the scaphoid and pisiform as landmarks.

Two [20,31] of the 12 studies used a speckle-tracking algorithm to evaluate the transverse displacement of the MN. In the Filius et al. [31] study, the MN was placed in a region of interest (ROI) in the first frame of the scan, and a speckle-tracking algorithm was used to calculate the total displacement. Kuo et al. [20] manually outlined the outermost hyperechoic rim of the MN and then used a multilevel BSP integrated algorithm for analysis. Among the articles targeting longitudinal MN gliding, two studies [30,33] used the duplex Doppler waveform method, and the other three studies [29,31,32] adopted a speckle-tracking algorithm to calculate total longitudinal gliding.

In addition to heterogeneity between study designs, the variability of measurements might also contribute to conflicting outcomes. Although template matching–based CC and speckle tracking algorithms can automatically assess the similarity of speckle patterns from the target and compare it to the ROI in medical image tracking applications, most US images have speckle noise that may cause false tracking results [57,58]. Moreover, these methods are more unstable in tracking gliding along the longitudinal axis than in the transverse plane because the longitudinal shifting of the MN and flexor tendons is less clearly visible.

Duplex Doppler is an alternative way to track longitudinal MN gliding. However, it is unable to capture out-of-plane movements caused by transverse movement beyond the beam width. Moreover, spectral broadening is an important artifact in pulsed-wave Doppler ultrasonographic imaging; therefore, the results might overestimate the MN excursions if the intrinsic spectral broadening was not accurately corrected [30]. Thus, previous studies used the ratio of nerve excursion to tendon excursion to correct this factor [30,33]. To explore and quantify the discrepancy caused by different measurement methods, we recommend further studies to compare the MN gliding detected by duplex Doppler and CC or BSP.

### 4.5. Limitations

This systematic review had some limitations. First, the selection criteria strictly limited the search to comparative case–control studies; other study types, including reviews, systematic reviews, and randomized controlled trials, were excluded. Thus, this systematic review’s level of evidence is only 3A. Second, because there was a great deal of variety in finger and wrist motions, joint positions, and outcome parameters among the included studies, the results of the meta-analyses in the transverse plane showed high heterogeneity. Third, five studies were excluded from the meta-analysis for the transverse plane because the presented data could not be pooled and analyzed with those of the other studies.

## 5. Conclusions

This is the first systematic review and meta-analysis that investigated the difference between transverse and longitudinal MN displacements between CTS and healthy subjects. We enrolled 15 comparative case–control studies and included a total of 513 electrodiagnostic-confirmed CTS wrists and 433 healthy wrists, showing the high credibility of this study.

Despite the large heterogeneity among the included studies, our systemic review and meta-analysis supported that patients with CTS had less transverse and longitudinal MN displacement than healthy populations. Moreover, five of the 15 included studies reported that the decrease in transverse or longitudinal MN displacement in CTS was correlated with clinical symptoms or NCS severity. Further studies using standardized protocol to evaluate MN displacement and its relationship with clinical symptoms are needed before decreased MN displacement could be used as one of the diagnostic criteria for CTS.

## Figures and Tables

**Table 1:** Study characteristics.

Study	Wrists (CTS/Control)	Age (mean ± SD)	Gender (M, F)	Measuring Site (Transverse/Longitudinal)	US Image Analysis	Postures	Outcome
Nakamichi et al. [37]	30/30	54.15 ± 2.02	female only	proximal CT	Initial and final recorded frames of motion cycle	Passive/2nd finger/PIP and DIP joints/full flexion-extension	Transverse MN displacement
Erel et al. [29]	17/19	42.95 ± 9.56	5, 31	proximal CT/5–15 cm from the distal wrist crease	Transverse plane: Initial and final recorded frames of motion cycle Longitudinal plane: CC algorithm	Passive/MCP joint/90 degree flexion to neutral	Longitudinal MN gliding Transverse displacement of radial nerve, CSA, and AR
Hough et al. [30]	19/37	51.19 ± 12.62	16, 40	lunate-capitate intercarpal joint	Duplex Doppler	Active/all fingers/full flexion-extension	Longitudinal MN and FDS gliding with elbow flexion and extension
van Doesburg et al. [38]	29/29	43.3 ± 13.59	25, 33	proximal CT	Initial and final recorded frames of motion cycle	Active/ 4 motions: 1st; 2nd; 3rd finger flexion; full flexion of four fingers	Transverse MN displacement along DP and RU axis, the distance between MN and the tendons
Yoshii et al. [39]	51/62	51.39 ± 13.85	11, 48	proximal CT	Initial and final recorded frames of motion cycle	Active/all fingers/full extension to flexion	Transverse MN displacement along DP and RU axis, CSA, perimeter, AR, and circularity
Wang et al. [36]	20/20	45.77 ± 8.55	10, 13	proximal CT	Initial and final recorded frames of motion cycle	Active/6 motions: finger flexion, wrist flexion with fingers extended, wrist flexion with fingers flexed, wrist extension with fingers extended, wrist extension with fingers flexed, and wrist ulnar deviation with fingers extended	Transverse DR and MN displacement (described as vector and magnitude) normalized to hand length (tip of middle finger to midline of distal wrist crease)
Nanno et al. [41]	21/21	69 ± 12.25	5, 16	proximal CT	Initial and final recorded frames of motion cycle	Active/all five fingers/with 5 wrist position (neutral, dorsal flexion, palmar flexion, ulnar deviation and radial deviation)/full extension-flexion	Transverse RDR and DDR
Filius et al. (a) [31]	113/42	41.71 ± 12.18	41, 114	proximal CT	Speckle-tracking algorithm	Active/all five fingers/full flexion in 8 s	Longitudinal gliding of MN, FDS3, FDP3, SSCT, displacement ratios of the MN and tendons Transverse plane: area, perimeter, circularity, DR, CoM
Filius et al. (b) [32]	25/14	46.5 ± 12.64	13, 19	proximal CT l	Speckle-tracking algorithm	Active/all five fingers/full extension to flexion/within 8, 4, and 2 s	Longitudinal gliding of MN, FDS3, FDP3 in low, medium, and high velocity
Kuo et al. [20]	40/32	-	-	proximal CT	Speckle-tracking algorithm (BSP)	Active/all five fingers/flexion- extension cycles	Transverse plane: R square, curvature, amplitude of MN displacement
Kang et al. [42]	22/23	55.82 ± 2.30	female only	proximal CT	Initial and final recorded frames of motion cycle	Active/4 motions: First, second, third finger full flexion and grip motion	Transverse MN displacement along DP and RU axis
Park et al. [43]	39/13	60.50 ± 11.57	15, 22	proximal CT	Initial and final recorded frames of motion cycle	Active/ Maximal voluntary motion of 6 motions (1st, 2nd, 3rd finger flexion, grasp, wrist ulnar deviation with finger extension, wrist radial deviation with finger extension)	Transverse MN MCV and relative to wrist width, CSA, and AR
Fan et al. [44]	16/16	61.29 ± 13.71	14, 18	proximal CT and mid-forearm	Initial and final recorded frames of motion cycle	Active/all five fingers/full extension to flexion	Transverse plane: echo intensity of the paraneural area, MN and myofascial structure; MN displacement
Liu et al. [33]	49/48	49.70 ± 9.46	11, 86	pisiform level/lunate- capitate intercarpal joint	Duplex Doppler	Active/2nd finger/MCP and proximal IP joints/full extension to flexion/in the neutral and 30 degree extension of wrist/at speed of 1 cycle per sec	Longitudinal MN gliding Transverse MN CSA, FR
Hara et al. [54]	6/6	68.42 ± 11.88	1, 11	tubercle of Tm	Composite image created from audio-video interleave file	Passive/ wrist joint/ neutral to full palmar flexion	Transverse plane: CSA, MAMn, RMMn, MR

-: not available. Abbreviations: US = ultrasound; MN = median nerve; CT = carpal tunnel; CSA = cross-sectional area; FR = flattening ratio; AR = aspect ratio; DR = deformation ratio; RDR = radial deviation ratio; DDR = dorsal deviation ratio; DP = dorsopalmar; RU = radioulnar; FDS = flexor digitorum superficialis; FDP = flexor digitorum profundus; SSCT = subsynovial connective tissue; BSP: multilevel block-sum pyramid integrate algorithm; CoM = center of mass; MCV = maxmal change value; MAMn = motion area of the MN; RMMn = real motion area of MN; MR = mobile ratio; CC: Cross-correlation algorithm; Tm = trapezium.

**Table 2:** Risk bias assessment by the Newcastle–Ottawa Quality Assessment Scale (NOS).

Study	Selection				Comparability Control for Important Factor	Exposure			Score
	Adequate Definition of Cases	Representativeness of the Cases	Selection of Controls	Definition of Controls		Ascertainment of Exposure	Same Method of Ascertainment for Cases & Controls	Non-Response Rate	
Nakamichi et al. [37]	✩	✩	✩	✩	✩✩	✩	✩	✩	9
Erel et al. [29]	✩	✩	✩	✩	✩✩	-	✩	✩	8
Hough et al. [30]	✩	✩	✩	✩	✩	✩	✩	✩	8
van Doesburg et al. [38]	✩	✩	✩	✩	✩	-	✩	✩	7
Yoshii et al. [39]	✩	✩	✩	✩	✩	-	✩	✩	7
Wang et al. [36]	✩	✩	✩	✩	✩	-	✩	✩	7
Nanno et al. [41]	✩	✩	✩	✩	✩	-	✩	✩	7
Filius et al. (a) [31]	✩	✩	✩	✩	✩	-	✩	✩	7
Filius et al. (b) [32]	✩	✩	✩	✩	✩	-	✩	✩	7
Kuo et al. [20]	✩	✩	✩	✩	✩	-	✩	✩	7
Kang et al. [42]	✩	✩	✩	✩	✩✩	-	✩	✩	8
Park et al. [43]	✩	✩	✩	✩	✩	-	✩	✩	7
Fan et al. [44]	✩	✩	✩	✩	✩✩	-	✩	✩	8
Liu et al. [33]	✩	✩	✩	✩	✩	✩	✩	✩	8
Hara et al. [54]	✩	✩	✩	✩	✩	-	✩	✩	7

The NOS is a star system (0 to 9 stars) for judging eight items across three perspectives. In the selection section, studies with adequate definitions for cases and controls are awarded a star for each item. In the comparability section, studies receive 1 to 2 stars if confounding factors are well controlled. In the exposure section, studies with secure records, blinded interviews, and the same response rate or enrollment methods between groups receive one star per item.

**Table 3:** Synthesis of results, transverse median nerve displacement.

Study	CTS (Mean ± SD)	Control (Mean ± SD)	*p* Value
Nakamichi et al. [37]			
Index finger flexion (mm)	0.37 ± 0.34	1.75 ± 0.49	0.0001
Erel et al. [28]	RU	RU	
MCP joint flexion (mm)	0.89 ± 1.15	1.55 ± 1.04	>0.08
van Doesburg et al. [38]	DP*; RU*	DP*; RU*	
First finger flexion (mm)	−0.10 ± 0.21;-0.63 ± 0.76	0.02 ± 0.23; 0.17 ± 0.84	<0.05; <0.05
Second finger flexion (mm)	0.13 ± 0.31; 1.25 ± 1.43	0.04 ± 0.35; 0.49 ± 1.61	>0.05; <0.038
Third finger flexion (mm)	0.19 ± 0.33; 1.90 ± 1.64	0.09 ± 0.38; 1.13 ± 2.13	>0.05; <0.0001
Four fingers flexion (mm)	0.09 ± 0.39; 1.63 ± 2.29	0.18 ± 0.39; 1.40 ± 1.95	>0.05; >0.05
Yoshii et al. [39]	DP*; RU*	DP*; RU*	
All fingers flexion (mm)	0.069 ± 0.438; 2.05 ± 2.82	0.377 ± 0.399; 2.45 ± 1.76	0.06; <0.01
Wang et al. [36]			
Fingers flex. with neutral wrist postion (NU)	Vector; Magnitude	Vector; Magnitude	
Wrist flex. with fingers ext. (NU)	0.1; 0.75 ± 0.44	0.2; 0.82 ± 0.33	0.217; >0.05
Wrist flex. with fingers flex. (NU)	0.8; 1.74 ± 0.78	1.5; 2.36 ± 0.79	<0.01; 0.016
Wrist ext. with fingers ext. (NU)	1.0; 1.71 ± 0.90	1.8; 2.46 ± 0.84	<0.01; 0.010
Wrist extension with fingers flexed. (NU)	0.2; 0.90 ± 0.68	0.4; 0.77 ± 0.46	<0.05; >0.05
Wrist UD with fingers ext. (NU)	0.6; 0.85 ± 0.56	0.5; 0.81 ± 0.58	0.106; >0.05
(normalized to hand length; 1NU = 1.8 mm)	1.8; 1.93 ± 1.23	2.8; 2.86 ± 0.51	<0.01; 0.005
Nanno et al. [41]			<0.05
All fingers flexion,	RDR; DDR	RDR; DDR	
with neutral wrist position	ext.: 3.54 ± 0.51; 6.43 ± 1.37; flex.: 4.81 ± 0.64: 5.42 ± 0.86	ext.: 7.89 ± 0.84; 9.09 ± 0.92; flex.: 9.75 ± 0.84: 7.53 ± 0.68	
wrist dorsal flexion	ext.: 4.77 ± 1.04; 7.8 ± 1.06; flex.: 7.02 ± 1.56: 6.46 ± 4.61	ext.: 8.56 ± 0.68; 10.42 ± 1.42; flex: 10.37 ± 1.34: 8.54 ± 1.72	
wrist palmar flexion	ext.: −7.66 ± 2.47; 1.56 ± 1.44; flex.: −10.61 ± 2.7: 0.52 ± 1.32	ext.: −1.54 ± 0.85; 6.54 ± 1.18; flex.: −6.98 ± 1.76: 4.8 ± 2.01	
wrist ulnar deviation	ext.: 6.34 ± 1.69; 5.2 ± 0.96; flex.: 8.99 ± 2.53: 4.57 ± 3.95	ext.: 9.32 ± 0.79; 8.26 ± 1.1; flex.: 12.2 ± 1.71: 7.03 ± 0.9	
wrist radial deviation	ext.: −2.11 ± 1.37; 2.31 ± 1.18; flex.: −3.09 ± 0.92: 0.97 ± 1.27	ext.: −0.65 ± 0.48; 7.73 ± 1.43; flex.: −2.19 ± 0.96: 5.95 ± 1.33	
Filius et al. (a) [31]	relative to FDS3	relative to FDS3	
All fingers flexion	-	-	>0.05
Kang et al. [42]	DP; RU	DP; RU	
First finger flexion (mm)	0.22 ± 0.07; 0.29 ± 0.08	0.32 ± 0.06; 0.81 ± 0.18	0.195; 0.012
Second finger flexion (mm)	0.30 ± 0.10; 0.40 ± 0.12	0.50 ± 0.10; 0.98 ± 0.21	0.099; 0.013
Third finger flexion (mm)	0.36 ± 0.11; 0.55 ± 0.16	0.95 ± 0.14; 1.05 ± 0.27	0.017; 0.195
Grip (mm)	0.29 ± 0.08; 0.40 ± 0.13	0.64 ± 0.11; 0.84 ± 0.18	0.015; 0.021
Kuo et al. [20] All five fingers flexion	R square; curvature; amplitude mild CTS: 0.77 ± 0.15; 0.25 ± 0.23; 0.57 ± 0.42 severe CTS: 0.56 ± 0.19; 0.12 ± 0.11; 0.35 ± 0.31	R square; curvature; amplitude 0.94 (0.02); 0.69 (0.28); 1.27 (0.62)	<0.001; <0.001; <0.001
Park et al. [43] MCV (mm; NU%)	Bland grade 1: 0.51 ± 0.17; 15.56 ± 5.08 Bland grade 2: 0.45 ± 0.09; 14.47 ± 3.03 Bland grade 3: 0.25 ± 0.08; 7.20 ± 2.19	0.5 ± 0.10; 15.27 ± 3.49	>0.05; >0.05 >0.05; >0.05 <0.05; <0.001
Fan et al. [44]			
All five fingers flexion (proximal CT, mm)	0.704 ± 0.159	2.346 ± 0.826	<0.01
All five fingers flexion (mid-forearm, mm)	0.808 ± 0.242	2.050 ± 0.873	<0.01
Hara et al. [54]			
Wrist flexion (MAMn, mm^2^)	11.8 ± 4.23	23.1 ± 4.28	<0.01
Wrist flexion (RMMn, mm^2^)	5.35 ± 2.32	16.35 ± 4.16	<0.01
Wrist flexion (MR)	1.86 ± 0.27	3.52 ± 0.79	<0.01

-: not available. Abbreviations: DP* = dorsal (−) palmar (+) axis; RU* = radial (−) palmar (+) axis; V = vector; M = magnitude; RDR = radial deviation ratio; DDR = dorsal deviation ratio; UD = ulnar deviation; NU = normalized unit; ext. = extension; flex. = flexion; MCV = maximal change value; Proximal CT = proximal carpal tunnel; MAMn = motion area of the MN; RMMn = real motion area of MN; MR = mobile ratio.

**Table 4:** Synthesis of results, longitudinal median nerve gliding.

Studies Using Speckle Tracking Method	CTS	Control	*p* Value
Erel et al. [29]			
passive MCP from 90 degree flex. to neutral (mm)	2.2 ± 0.93	2.62 ± 0.73	>0.1
Filius et al. (a) [31]			
all fingers flex. (clinical grading, mm)	minimal: 3.9 ± 1.2; mild: 3.1 ± 1.6; moderate: 2.7 ± 1.5; severe: 3.1 ± 1.5	4.1 ± 1.9	0.019 (mild/moderate v.s control)
all fingers flex. (NCS grading, mm)	minimal: 3.1 ± 1.5; mild: 3.4 ±1.7; moderate: 2.6 ± 1.4; severe: 2.4 ± 1.0	4.1 ± 1.8	0.001 (moderate/severe v.s control)
Filius et al. (b) [32]			
all fingers flex. (low/medium/high motion velocity, cm)	0.18 ± 0.10; 0.21 ± 0.12; 0.23 ± 0.12	0.21 ± 0.07; 0.33 ± 0.10; 0.40 ± 0.11	< 0.001; 0.002; <0.001
all fingers flex. (low/medium/high motion velocity, NU*)	0.10 ± 0.06; 0.12 ± 0.07; 0.13 ± 0.08	0.22 ± 0.07; 0.23 ± 0.07; 0.28 ± 0.11	<0.001; <0.001; <0.001
**Studies using Duplex Doppler waveform**	**CTS**	**Control**	***p* value**
Hough et al. [30]			
all fingers flex. with elbow flexion & extension (mm)	10.2 ± 3.1; 8.3 ± 2.6	12.5 ± 2.5; 11.2 ± 2.8	0.089; 0.013
all fingers flex. with elbow flexion & extension (NU*)	0.28 ± 0.10; 0.23 ± 0.06	0.36 ± 0.06; 0.32 ± 0.07	0.019; <0.001
Liu et al. [33] index finger flex. (neutral; 30 degree ext. wrist, mm) index finger flex. (neutral; 30 degree ext. wrist, NU*)	18.5 ± 7.0; 21.3 ± 10.6 0.20 ± 0.11; 0.21 ± 0.11	23.7 ± 9.1; 25.6 ± 10.3 0.29 ± 0.15; 0.29 ± 0.14	0.0001; 0.02 0.0008; 0.005

Abbreviation: MCP = meta-carpophalangeal joint; NU*= MN relative to FDS displacement; ext.= extension; flex. = flexion; CTS = carpal tunnel syndrome.

## Data Availability

The data analysed in this systematic review are included in this article. Further datasets used and/or analysed in this study are available from the corresponding author on request. The data are not publicly available due to proprietary nature.

## Supplementary Materials

The following supporting information can be downloaded at: https://www.mdpi.com/article/10.3390/diagnostics12102349/s1.

## Abbreviations

CTS	carpal tunnel syndrome
MN	median nerve
US	ultrasound
SSCT	subsynovial connective tissue
FDS	flexor digitorum superficialis
FDP	flexor digitorum profundus
FPL	flexor pollicis longus
MCV	maximal change value
Tm	trapezium
NCSs	nerve conduction studies
EMG	electromyography
CSA	cross-sectional area
DCT	the depth of the carpal tunnel
WFR	wrist-forearm ratio
WFD	wrist-forearm difference
MUR	median-to-ulnar nerve ratio
MUD	median-to-ulnar nerve difference
DP	dorsopalmar
RU	radioulnar
DDR	radial deviation ratio
RDR	dorsal deviation ratio
MAMn	the summed motion area of the MN
RMMn	real motion area
MR	motion ratio
BSP	multilevel block-sum pyramid integrate algorithm
CC	cross-correlation algorithm
NOS	Newcastle–Ottawa Quality Assessment Scale
VTI	velocity-time integral
ROI	region of interest
CoM	center of mass
