# Supplementary material for: Ultrasonographical Evaluation of the Median Nerve Mobility in Carpal Tunnel Syndrome: A Systematic Review and Meta-Analysis
Source: Diagnostics (Basel). 2022 Sep 28;12(10):2349. doi: 10.3390/diagnostics12102349 (PMC9600711; doi:10.3390/diagnostics12102349)
Supplement: Supplementary file 1 [file diagnostics-12-02349-s001.zip › 0814 Supplementary file, S1 (keywords).pdf]

Supplementary data S1. Keywords that were used for searching in three databases.

PubMed

((nerve sliding) OR (nerve gliding) OR (nerve excursion) OR (nerve deformation) OR (nerve displacement) OR (nerve movement)) AND "median nerve" AND ("Ultrasonography"[Mesh] OR "Ultrasonics"[Mesh]) AND "Carpal Tunnel Syndrome"[Mesh]

EMBASE

((("nerve".af or "nerve s".af or "nerved".af or "nerves".af) and ("slide".af or "slides".af or "sliding".af or "slidings".af)) or (("nerve".af or "nerve s".af or "nerved".af or "nerves".af) and ("glide".af or "glided".af or "glides".af or "gliding".af)) or (("nerve".af or "nerve s".af or "nerved".af or "nerves".af) and ("excursion".af or "excursions".af)) or (("nerve".af or "nerve s".af or "nerved".af or "nerves".af) and ("abnormalities".fs or "abnormalities".af or "deformities".af or exp "congenital abnormalities"/ or ("congenital".af and "abnormalities".af) or "congenital abnormalities".af or "deformity".af or "deform".af or "deformabilities".af or "deformability".af or "deformable".af or "deformably".af or "deformation".af or "deformational".af or "deformations".af or "deformative".af or "deformed".af or "deforming".af or "deforms".af)) or (("nerve".af or "nerve s".af or "nerved".af or "nerves".af) and ("displace".af or "displaced".af or exp "displacement, psychological"/ or ("displacement".af and "psychological".af) or "psychological displacement".af or "displacement".af or "displacements".af or "displaces".af or "displacing".af)) or (("nerve".af or "nerve s".af or

"nerved".af or "nerves".af) and (exp "movement"/ or "movement".af or "movements".af or "movement s".af))) and "median nerve".af and (exp "ultrasonography"/ or exp "ultrasonics"/) and exp "carpal tunnel syndrome"/

Cochrane library

((nerve sliding) OR (nerve gliding) OR (nerve excursion) OR (nerve deformation) OR (nerve displacement) OR (nerve movement)) AND “median nerve" AND ("Ultrasonography" OR "Ultrasonics") AND "Carpal Tunnel Syndrome"
