# Supplementary material for: Ultrasonographical Evaluation of the Median Nerve Mobility in Carpal Tunnel Syndrome: A Systematic Review and Meta-Analysis
Source: Diagnostics (Basel). 2022 Sep 28;12(10):2349. doi: 10.3390/diagnostics12102349 (PMC9600711; doi:10.3390/diagnostics12102349)
Supplement: Supplementary file 1 [file diagnostics-12-02349-s001.zip › 0922 Supplementary figures.pdf]

Appendices

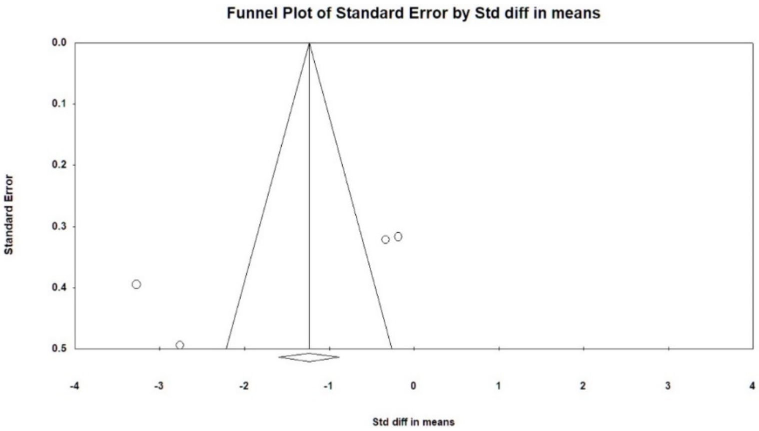

Supplementary Figure S1. Funnel plots of studies investigating actual transverse MN displacement.

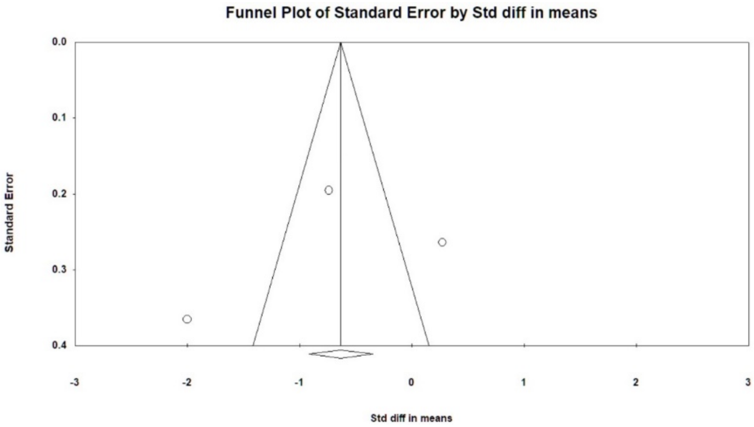

Supplementary Figure S2. Funnel plots of studies investigating transverse MN displacement at DP axis.

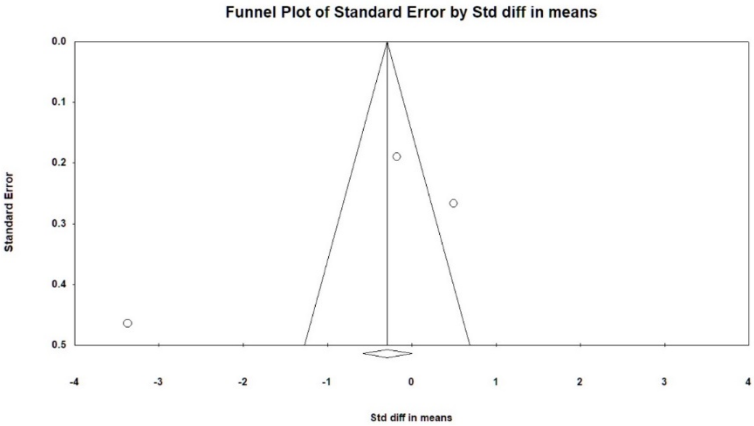

Supplementary Figure S3. Funnel plots of studies investigating transverse MN displacement at RU axis.

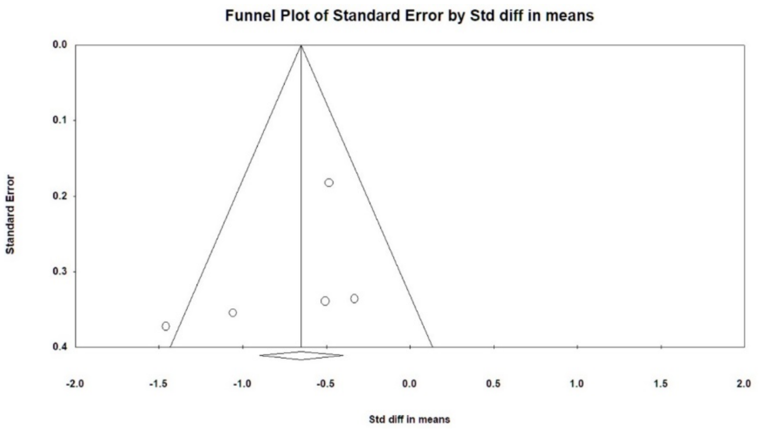

Supplementary Figure S4. Funnel plots of studies investigating longitudinal MN gliding, speckle tracking method.

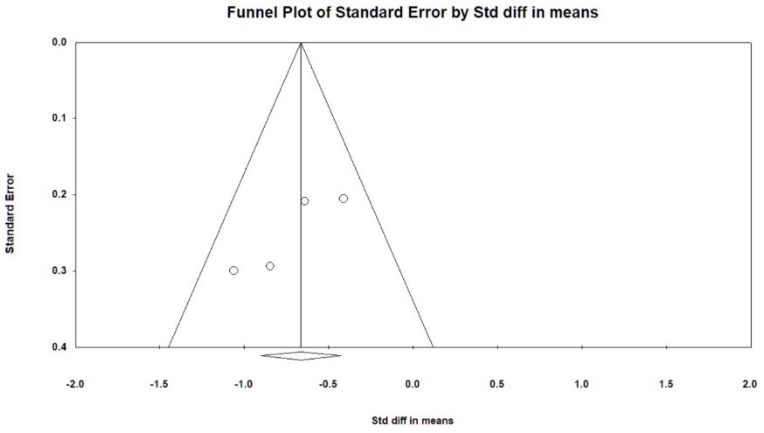

Supplementary Figure S5. Funnel plots of studies investigating longitudinal MN gliding, duplex Doppler waveform method.

Study name	Statistics with study removed						
	Point	Standard error	Variance	Lower limit	Upper limit	Z-Value	p-Value
Nakamichi et al., 1995	-1.039	0.695	0.483	-2.400	0.323	-1.495	0.135
Wang et al., 2014	-2.105	1.007	1.014	-4.079	-0.131	-2.091	0.037
Park et al., 2017	-2.055	1.065	1.134	-4.142	0.032	-1.930	0.054
Fan et al., 2020	-1.248	0.929	0.863	-3.069	0.572	-1.344	0.179
	-1.612	0.796	0.634	-3.173	-0.051	-2.025	0.043

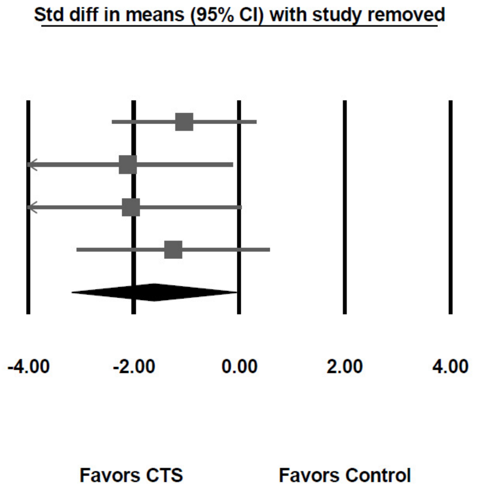

Supplementary Figure S6. The sensitivity analysis of studies evaluating actual transverse MN displacement.

Study name	Statistics with study removed						
	Point	Standard error	Variance	Lower limit	Upper limit	Z-Value	p-Value
Erel et al., 2003	-0.959	0.844	0.713	-2.614	0.695	-1.136	0.256
van Doesburg et al., 2012	-1.336	0.830	0.689	-2.963	0.291	-1.610	0.107
Yoshii et al., 2013	-1.128	1.030	1.060	-3.146	0.890	-1.096	0.273
Kang et al., 2016	-0.071	0.288	0.083	-0.635	0.493	-0.247	0.805
	-0.855	0.619	0.383	-2.068	0.358	-1.382	0.167

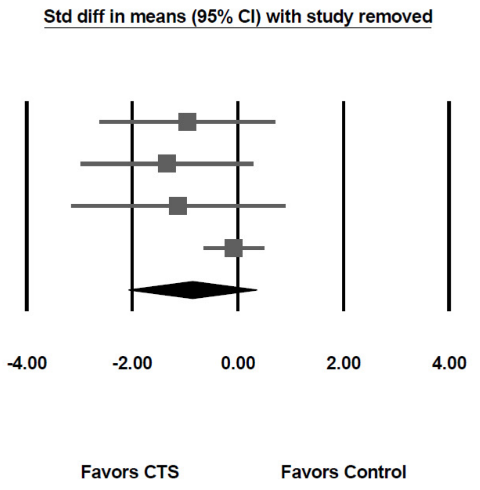

Supplementary Figure S7. The sensitivity analysis of studies evaluating transverse MN displacement at DP axis.

Study name	Statistics with study removed						
	Point	Standard error	Variance	Lower limit	Upper limit	Z-Value	p-Value
van Doesburg et al., 2012	-1.332	0.630	0.396	-2.565	-0.098	-2.115	0.034
Yoshii et al., 2013	-0.850	1.136	1.291	-3.076	1.377	-0.748	0.454
Kang et al., 2016	-0.249	0.505	0.255	-1.239	0.741	-0.492	0.622
	-0.795	0.556	0.310	-1.886	0.295	-1.430	0.153

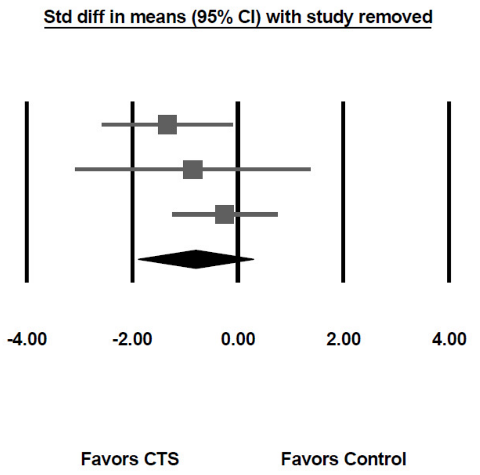

Supplementary Figure S8. The sensitivity analysis of studies evaluating transverse MN displacement at RU axis.

Study name	Statistics with study removed						
	Point	Standard error	Variance	Lower limit	Upper limit	Z-Value	p-Value
Erel et al.	-0.712	0.120	0.014	-0.947	-0.478	-5.954	0.000
Filius et al.(a)	-0.738	0.125	0.016	-0.982	-0.494	-5.929	0.000
Filius et al.(b), low velocity	-0.723	0.115	0.013	-0.949	-0.497	-6.277	0.000
Filius et al.(b), medium velocity	-0.659	0.113	0.013	-0.879	-0.438	-5.852	0.000
Filius et al.(b), high velocity	-0.611	0.089	0.008	-0.787	-0.436	-6.836	0.000
Hough et al., all fingers flexion with elbow flexion	-0.677	0.120	0.014	-0.913	-0.442	-5.634	0.000
Hough et al., all fingers flexion with elbow extension	-0.645	0.111	0.012	-0.862	-0.428	-5.830	0.000
Liu et al., index finger flexion with neutral wrist position	-0.709	0.129	0.017	-0.962	-0.457	-5.502	0.000
Liu et al., index finger flexion with 30 degree wrist extension	-0.741	0.118	0.014	-0.974	-0.509	-6.262	0.000
	-0.689	0.109	0.012	-0.902	-0.476	-6.337	0.000

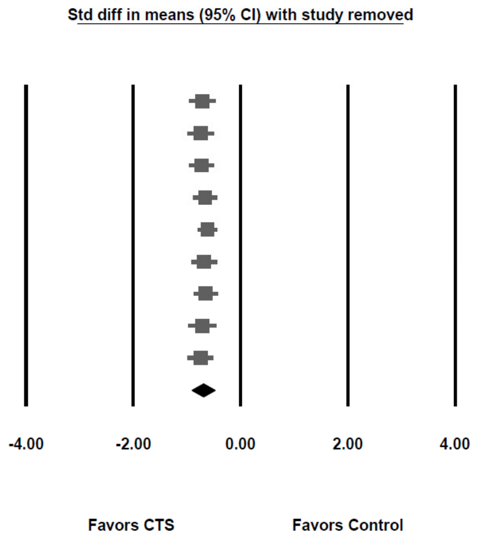

Supplementary Figure S9. The sensitivity analysis of studies evaluating longitudinal gliding.
